# Supplementary material for: Modeling glioblastoma heterogeneity as a dynamic network of cell states
Source: Mol Syst Biol. 2021 Sep 16;17(9):e10105. doi: 10.15252/msb.202010105 (PMC8444284; doi:10.15252/msb.202010105)
Supplement: Supplementary file 5 — Source Data for Figure 3 [file MSB-17-e10105-s001.zip › Figure3A_sourcedata/GSEA_3065/hallmarks_state3.GseaPreranked.1623416274744/HALLMARK_TNFA_SIGNALING_VIA_NFKB.html]

Details for gene set HALLMARK\_TNFA\_SIGNALING\_VIA\_NFKB[GSEA]

|  || Dataset | state3 |
| Phenotype | NoPhenotypeAvailable |
| Upregulated in class | na\_neg |
| GeneSet | HALLMARK\_TNFA\_SIGNALING\_VIA\_NFKB |
| Enrichment Score (ES) | -0.29691023 |
| Normalized Enrichment Score (NES) | -1.0528442 |
| Nominal p-value | 0.3703125 |
| FDR q-value | 0.52589947 |
| FWER p-Value | 1.0 |
Table: GSEA Results Summary

  

Fig 1: Enrichment plot: HALLMARK\_TNFA\_SIGNALING\_VIA\_NFKB      
 Profile of the Running ES Score & Positions of GeneSet Members on the Rank Ordered List

  

| PROBE | GENE SYMBOL | GENE\_TITLE | RANK IN GENE LIST | RANK METRIC SCORE | RUNNING ES | CORE ENRICHMENT || 1 | TNFAIP6 |  |  | 158 | 0.307 | 0.0128 | No |
| 2 | TNC |  |  | 249 | 0.264 | 0.0293 | No |
| 3 | ATP2B1 |  |  | 271 | 0.253 | 0.0525 | No |
| 4 | IL6ST |  |  | 316 | 0.239 | 0.0717 | No |
| 5 | LDLR |  |  | 498 | 0.199 | 0.0710 | No |
| 6 | CCNL1 |  |  | 529 | 0.194 | 0.0872 | No |
| 7 | PTX3 |  |  | 605 | 0.185 | 0.0974 | No |
| 8 | PLPP3 |  |  | 786 | 0.159 | 0.0927 | No |
| 9 | JAG1 |  |  | 1011 | 0.138 | 0.0807 | No |
| 10 | SLC2A3 |  |  | 1026 | 0.136 | 0.0930 | No |
| 11 | FJX1 |  |  | 1071 | 0.133 | 0.1014 | No |
| 12 | SLC2A6 |  |  | 1120 | 0.128 | 0.1088 | No |
| 13 | PTPRE |  |  | 1396 | 0.109 | 0.0881 | No |
| 14 | IER5 |  |  | 1491 | 0.103 | 0.0877 | No |
| 15 | HES1 |  |  | 1501 | 0.103 | 0.0971 | No |
| 16 | VEGFA |  |  | 1723 | 0.090 | 0.0807 | No |
| 17 | CD44 |  |  | 1748 | 0.089 | 0.0869 | No |
| 18 | B4GALT5 |  |  | 1761 | 0.088 | 0.0945 | No |
| 19 | NFAT5 |  |  | 1995 | 0.076 | 0.0753 | No |
| 20 | SDC4 |  |  | 2189 | 0.069 | 0.0600 | No |
| 21 | ABCA1 |  |  | 2275 | 0.065 | 0.0568 | No |
| 22 | MYC |  |  | 2324 | 0.064 | 0.0577 | No |
| 23 | TAP1 |  |  | 2349 | 0.063 | 0.0613 | No |
| 24 | PDE4B |  |  | 2443 | 0.059 | 0.0565 | No |
| 25 | NAMPT |  |  | 2583 | 0.054 | 0.0459 | No |
| 26 | F2RL1 |  |  | 2594 | 0.054 | 0.0502 | No |
| 27 | BTG3 |  |  | 2610 | 0.053 | 0.0539 | No |
| 28 | KDM6B |  |  | 2805 | 0.047 | 0.0362 | No |
| 29 | REL |  |  | 2985 | 0.041 | 0.0197 | No |
| 30 | B4GALT1 |  |  | 3043 | 0.040 | 0.0172 | No |
| 31 | SERPINE1 |  |  | 3090 | 0.039 | 0.0158 | No |
| 32 | BCL6 |  |  | 3186 | 0.036 | 0.0085 | No |
| 33 | BIRC2 |  |  | 3207 | 0.035 | 0.0098 | No |
| 34 | IER2 |  |  | 3345 | 0.032 | -0.0028 | No |
| 35 | CSF1 |  |  | 3363 | 0.031 | -0.0016 | No |
| 36 | IRF1 |  |  | 3457 | 0.029 | -0.0094 | No |
| 37 | SMAD3 |  |  | 3767 | 0.021 | -0.0430 | No |
| 38 | MCL1 |  |  | 3848 | 0.019 | -0.0503 | No |
| 39 | FOS |  |  | 3899 | 0.018 | -0.0543 | No |
| 40 | TUBB2A |  |  | 4022 | 0.015 | -0.0670 | No |
| 41 | BTG2 |  |  | 4070 | 0.013 | -0.0711 | No |
| 42 | PLAUR |  |  | 4075 | 0.013 | -0.0702 | No |
| 43 | EGR1 |  |  | 4134 | 0.012 | -0.0757 | No |
| 44 | DUSP1 |  |  | 4186 | 0.010 | -0.0806 | No |
| 45 | MAP2K3 |  |  | 4277 | 0.007 | -0.0902 | No |
| 46 | PDLIM5 |  |  | 4368 | 0.005 | -0.1001 | No |
| 47 | JUNB |  |  | 4467 | 0.002 | -0.1113 | No |
| 48 | ZBTB10 |  |  | 4501 | 0.001 | -0.1149 | No |
| 49 | NFKB1 |  |  | 4528 | 0.001 | -0.1179 | No |
| 50 | EHD1 |  |  | 4716 | -0.004 | -0.1391 | No |
| 51 | DRAM1 |  |  | 4865 | -0.008 | -0.1554 | No |
| 52 | PPP1R15A |  |  | 4983 | -0.010 | -0.1679 | No |
| 53 | TRIP10 |  |  | 5239 | -0.017 | -0.1957 | No |
| 54 | BHLHE40 |  |  | 5331 | -0.019 | -0.2042 | No |
| 55 | SOCS3 |  |  | 5338 | -0.019 | -0.2030 | No |
| 56 | PLAU |  |  | 5363 | -0.020 | -0.2037 | No |
| 57 | ETS2 |  |  | 5490 | -0.024 | -0.2158 | No |
| 58 | KLF10 |  |  | 5514 | -0.025 | -0.2160 | No |
| 59 | KLF6 |  |  | 5627 | -0.028 | -0.2261 | No |
| 60 | NFKBIA |  |  | 5684 | -0.029 | -0.2296 | No |
| 61 | SNN |  |  | 5791 | -0.032 | -0.2385 | No |
| 62 | SOD2 |  |  | 5817 | -0.033 | -0.2381 | No |
| 63 | TNIP2 |  |  | 5869 | -0.035 | -0.2404 | No |
| 64 | ATF3 |  |  | 6159 | -0.045 | -0.2693 | No |
| 65 | RELA |  |  | 6181 | -0.046 | -0.2670 | No |
| 66 | BMP2 |  |  | 6331 | -0.050 | -0.2791 | No |
| 67 | DENND5A |  |  | 6405 | -0.053 | -0.2822 | No |
| 68 | TNIP1 |  |  | 6460 | -0.055 | -0.2829 | No |
| 69 | CFLAR |  |  | 6582 | -0.060 | -0.2908 | Yes |
| 70 | RIPK2 |  |  | 6615 | -0.062 | -0.2883 | Yes |
| 71 | GADD45B |  |  | 6619 | -0.062 | -0.2823 | Yes |
| 72 | RHOB |  |  | 6706 | -0.066 | -0.2855 | Yes |
| 73 | PLK2 |  |  | 6726 | -0.067 | -0.2809 | Yes |
| 74 | RCAN1 |  |  | 6813 | -0.072 | -0.2836 | Yes |
| 75 | CEBPB |  |  | 6863 | -0.074 | -0.2818 | Yes |
| 76 | TGIF1 |  |  | 6888 | -0.075 | -0.2769 | Yes |
| 77 | YRDC |  |  | 6926 | -0.077 | -0.2734 | Yes |
| 78 | FOSL2 |  |  | 6992 | -0.080 | -0.2728 | Yes |
| 79 | TANK |  |  | 6995 | -0.080 | -0.2649 | Yes |
| 80 | FOSL1 |  |  | 7031 | -0.083 | -0.2605 | Yes |
| 81 | TIPARP |  |  | 7051 | -0.084 | -0.2541 | Yes |
| 82 | DUSP4 |  |  | 7251 | -0.097 | -0.2673 | Yes |
| 83 | IRS2 |  |  | 7285 | -0.099 | -0.2611 | Yes |
| 84 | CCND1 |  |  | 7456 | -0.113 | -0.2693 | Yes |
| 85 | TSC22D1 |  |  | 7546 | -0.122 | -0.2672 | Yes |
| 86 | IFNGR2 |  |  | 7647 | -0.132 | -0.2654 | Yes |
| 87 | NFE2L2 |  |  | 7651 | -0.133 | -0.2523 | Yes |
| 88 | JUN |  |  | 7746 | -0.146 | -0.2484 | Yes |
| 89 | ID2 |  |  | 7934 | -0.174 | -0.2524 | Yes |
| 90 | LITAF |  |  | 8015 | -0.188 | -0.2426 | Yes |
| 91 | PNRC1 |  |  | 8133 | -0.212 | -0.2345 | Yes |
| 92 | CEBPD |  |  | 8158 | -0.217 | -0.2153 | Yes |
| 93 | NINJ1 |  |  | 8160 | -0.218 | -0.1933 | Yes |
| 94 | BTG1 |  |  | 8162 | -0.218 | -0.1712 | Yes |
| 95 | SQSTM1 |  |  | 8176 | -0.221 | -0.1503 | Yes |
| 96 | EIF1 |  |  | 8222 | -0.234 | -0.1318 | Yes |
| 97 | GADD45A |  |  | 8306 | -0.260 | -0.1151 | Yes |
| 98 | PMEPA1 |  |  | 8520 | -0.346 | -0.1046 | Yes |
| 99 | SAT1 |  |  | 8533 | -0.354 | -0.0701 | Yes |
| 100 | CDKN1A |  |  | 8657 | -0.461 | -0.0375 | Yes |
| 101 | PHLDA1 |  |  | 8670 | -0.476 | 0.0094 | Yes |
Table: GSEA details [plain text format]

  

Fig 2: HALLMARK\_TNFA\_SIGNALING\_VIA\_NFKB: Random ES distribution      
 Gene set null distribution of ES for **HALLMARK\_TNFA\_SIGNALING\_VIA\_NFKB**
